# Quality of life in endometrial cancer survivors by grade of disease

**DOI:** 10.1002/cam4.5987

**Published:** 2023-05-06

**Authors:** K. Banning, J. Fucinari, A. Fielder, J. J. Ruterbusch, J. L. Beebe‐Dimmer, A. G. Schwartz, J. J. Wallbillich, M. L. Cote

**Affiliations:** ^1^ Department of Obstetrics and Gynecology Wayne State University School of Medicine Detroit Michigan USA; ^2^ Department of Oncology Wayne State University School of Medicine Detroit Michigan USA; ^3^ Population Studies and Disparities Research Program The Barbara Ann Karmanos Cancer Institute Detroit Michigan USA; ^4^ Molecular Therapeutics Program The Barbara Ann Karmanos Cancer Institute Detroit Michigan USA

**Keywords:** endometrial cancer, quality of life, survivorship

## Abstract

**Background:**

Endometrial cancer (EC) is the most common gynecologic malignancy in developed countries, with overall incidence increasing, particularly high‐grade disease. There is sparse information regarding quality of life (QOL) in EC survivors with a focus on grade of disease.

**Methods:**

A total of 259 women with EC diagnosed between 2016 and 2020 were identified via the Metropolitan Detroit Cancer Surveillance System and consented to enroll in the Detroit Research on Cancer Survivors cohort study (if African American, *n* = 138) or completed the baseline interview (if non‐Hispanic white, *n* = 121). Each respondent provided information about their health history, educational attainment, health behaviors, and demographics. The Functional Assessment of Cancer Therapy‐General (FACT‐G) and Endometrial‐specific (FACT‐En) were used to assess QOL.

**Results:**

Women diagnosed with high‐grade (*n* = 112) and low‐grade (*n* = 147) EC participated in this study. EC survivors with high‐grade disease reported significantly lower QOL compared to survivors with low‐grade disease (85 vs. 91, respectively, *p* value = 0.025) as assessed by the FACT‐G. This difference was driven by lower physical and functional subscales among women with high‐grade disease compared to those with low‐grade disease (*p* value = 0.016 and *p* = 0.028, respectively). Interestingly, EC‐specific QOL measures, as assessed by the FACT‐En, did not differ by grade.

**Conclusion:**

Grade of disease impacts QOL in EC survivors, as well as socioeconomic, psychological, and physical factors. Most of these factors are amenable to interventions and should be assessed in patients after an EC diagnosis.

## 1 INTRODUCTION

Endometrial cancer (EC) is the most commonly diagnosed female gynecologic cancer in North America and Europe.^1^ Worldwide, cancers of the endometrium rank as the 6th most common malignancy in women, and are the 14th leading cause of death in women. The incidence and mortality rates of EC are rising, with 65,950 new diagnoses expected in 2022.^1^, ^2^, ^3^ Overall incidence of EC (all types) is highest in Black women, even after adjusting for hysterectomy, and Black women also have the highest mortality rates.^3^, ^4^, ^5^, ^6^ Historically, EC has been roughly dichotomized into type 1 and type 2 EC, with several histopathologic subtypes that are associated with different clinical outcomes.^7^ Although this classification system has limitations, high‐grade disease is usually classified as type 2 EC, is more aggressive, and is more commonly diagnosed in Black women.

Concerning cancer survivorship among women, EC survivors are only second in number to breast cancer survivors in the United States.^3^, ^6^, ^8^ Health related quality of life (QOL) in survivors of EC is becoming more important as incidence of EC and the number of survivors rise.^1^, ^2^, ^3^ Broadly defined by the Centers for Disease Control and Prevention as, “physical and mental health perceptions and their correlates, including health risks and conditions, functional status, social support, and socioeconomic status”, QOL is more often being recognized as an important patient‐reported outcome in addition to disease‐free or overall survival after a cancer diagnosis.^9^ Glasspool et al. recently reported findings regarding QOL in a large cohort of women with gynecologic cancers, including 811 women with EC, although results were not broken out by disease site.^10^ They reported higher depression and anxiety scores, as well as having two or more comorbid conditions were associated with lower QOL, and being over the age of 50, being physically active, as well as being obese were associated with higher QOL scores.^10^ As cancers are detected at earlier stages and more effective therapies become available, more people are living with cancer in maintenance or remission, potentially creating a challenge for those living with the effects of their diagnosis and treatment that could linger for decades.^11^ QOL after an EC diagnosis is a critical component to consider as most women live for years, if not decades, after diagnosis. Assessing QOL can give providers insight into the long‐term effects of cancer and its treatment as well as identify potentially modifiable factors to improve QOL. A systematic review of patient‐reported outcomes after an EC diagnosis did not report on any studies that included analyses of high‐ versus low‐grade disease, as most studies focus on QOL difference by treatment regimen.^12^

Due to the aggressive nature of high‐grade EC, we hypothesized that those with high‐grade disease tumors may report significantly lower QOL compared to women with low‐grade disease. We surmised this may be due to longer treatment protocols, more intense treatment regimens, and overall worse prognosis associated with high‐grade disease.^3^, ^6^ The goal of this study was to assess QOL in a population‐based cohort of EC survivors, and to examine factors associated with QOL among EC survivors, with a focus on grade.

## 2 METHODS

### 2.1 Study population

Women who were diagnosed with EC between 2016 and 2020 were identified via the Metropolitan Detroit Cancer Surveillance System (MDCSS) and invited to participate. The MDCSS is a population‐based cancer registry that captures incident cancers in residents of Wayne, Oakland, and Macomb counties in Southeastern Michigan. Women identified as African American or Black via medical records were invited to participate in the Detroit Research on Cancer Survivors (ROCS) study, a cohort study which just completed baseline recruitment of African Americans with lung, breast, prostate, colorectal, or EC, as well as early onset (under age 50 at diagnosis) cancers of any type. The identification and recruitment of non‐Hispanic white (NHW) women, who were not eligible for ROCS, to complete the baseline survey, was conducted under the same protocols in place for the ROCS study of African American women. Additionally, identification of NHW women with EC was limited to those diagnosed in 2016 and 2017, with oversampling of women with high‐grade disease. Details regarding recruitment have been published as supplementary material by Beebe‐Dimmer et al.^13^ Briefly, cancer survivors identified by MDCSS were sent an introductory letter at least 5 months post‐diagnosis, explaining the aims of the study. Included in this packet was a written version of the questionnaire (along with a postage‐paid return envelope), and information on how to complete the survey on‐line if they choose. If a survey response was not received after 3 weeks, the research team attempted to contact the survivor by phone to answer any questions they may have regarding the study and offered to complete the survey by phone (interviewer administered). As of March 2021, 816 EC survivors have been identified, 259 women had completed the survey with data available at the time of analysis. Recruitment for ROCS was ongoing through 1/31/2022. This study has been reviewed and approved by Wayne State University IRB (#050417M1F) and all participants provided informed consent.

### 2.2 Data collection

Clinical characteristics such as SEER summary stage, ICD‐O‐3 histology codes, and grade were obtained from MDCSS. Tumors were classified as either high grade or low grade using histology and grade information, with the following sites being classified as high grade: Clear cell (ICD code: 8310), serous (ICD code: 8441, 8460, 8461), and carcinosarcoma (ICD codes: 8950, 8980, 8951, 8981). In addition, endometrioid (ICD codes: 8050, 8140, 8143, 8210, 8211, 8260, 8261, 8262, 8263, 8340, 8380, 8381, 8382, 8383, 8384, 8560, 8570) and mixed (ICD codes: 8323, 8255) tumors with grade 3 or 4 were classified as high grade. Endometrioid and mixed tumors with grade 1 or 2 were classified as low grade. All other histology codes (mainly leiomyosarcomas and stromal sarcomas) were excluded from this analysis (*n* = 8) due to the rarity of the diagnosis. Women self‐reported treatment for EC (surgery, radiation, hormone therapy, immunotherapy, chemotherapy), whether they were currently undergoing treatment, as well as their current body weight and weight 1 year prior to diagnosis. Additionally, history of various comorbid conditions was also collected via self‐report, including diabetes, hypertension, stroke, high cholesterol, chronic obstructive lung disease (COPD) or emphysema, arthritis, thyroid disease, and depression. Women also reported their smoking status (never, former, current), alcohol use in the last month (yes/no) and whether or not they were physically active in the last month using the International Physical Activity Questionnaire Short Form validated self‐reported scale to assess physical activity.^14^

Two standardized instruments were also utilized to assess QOL. First, the Functional Assessment of Cancer Therapy‐General (FACT‐G), a 27‐item questionnaire was utilized to measure physical, social, emotional, and functional well‐being that has been validated among cancer survivors with various types of cancer and in use for over two decades.^15^, ^16^ Second, the Functional Assessment of Cancer Therapy‐Endometrial cancer, a 43‐item questionnaire (FACT‐En), specifically developed for women with EC, was also administered. These questions were specific to concerns that may be more common after treatment for reproductive cancers, such as, “I have vaginal bleeding or spotting”. All FACT scores have a 5 point Likert‐type response scale, and were scored in accordance with guidelines provided by factit.org, with high scores representative of better QOL.^15^ Cronbach's alpha, a measure of internal consistency, was calculated.^17^ Minimal clinically important differences, a measure to identify differences likely to be meaningful to patients and clinicians as described by Yost and Eton, were also discussed.^18^ Briefly, an overall difference of 3–7 points and a sub score difference of 2–3 points would be considered a minimal clinically important difference.

### 2.3 Statistical analysis

All analyses were conducted using SAS statistical software version 9.4 (Cary) and graphs were drawn using R software.^19^ An alpha of 0.05 was set as a threshold of statistical significance. The distribution of demographic, treatment, medical history, and health behavior variables were compared by grade category using chi‐square tests. The median and range was calculated for FACT‐G total, the FACT‐G subscales (physical, social, emotional, and functional well‐being), the FACT‐En Trial Outcome Index, and FACT‐En Total. As the FACT‐G subscales were not normally distributed, a Wilcoxon rank sum test was used to compared the scores by grade category. The FACT‐G total score was normally distributed, and linear regression was used to examine the relationship between demographic and clinical variables with FACT‐G total. Univariate estimates were calculated for all variables, and a multivariable model was created that included variables significant at *p* < 0.05 in univariate analyses.

## 3 RESULTS

Demographic information collected from study participants is shown in Table 1, stratified by tumor grade, with interviews occurring approximately 2 years after diagnosis for all women regardless of grade. Women with high‐grade disease were older at diagnosis compared to women with low‐grade disease (median age in years of 65 compared to 61, respectively, *p* value<0.001). Due to the oversampling of NHW women with high‐grade cancers, there were no differences in grade by race, nor were differences seen by education level, marital status, or income. Table 1 also presents stage at diagnosis and treatment information. Women with low‐grade disease were more likely to be diagnosed with local disease compared to women with high‐grade disease (*p* value<0.001), thus women with high‐grade disease were more likely to receive radiation and chemotherapy (*p* value<0.001 for both). No differences by grade were identified for surgical treatment, with nearly all women reporting having surgery as part of their primary treatment. Hormone therapy and immunotherapy were rarely reported and also did not differ by grade. The vast majority of women were not currently in treatment at the time of study enrollment (93% of low grade and 92% of high grade). Lastly, the vast majority of women responded via written questionnaire as their preferred method of survey completion. There were no significant differences between survey method of completion and FACT‐G (*p* value = 0.125) or FACT‐En (*p* value = 0.070) or grade (*p* value = 0.139) (data not shown). Furthermore, the addition of survey method to the final multivariable model did not change the beta estimates for any of the significant findings.

**TABLE 1:** Select clinical and demographic variables for women diagnosed with endometrial cancer by grade category.

	Low grade		High grade		*p‐*value
	*N*	Row %	*N*	Row %	
Total	147	57%	112	43%	
*Demographics*					
Race					0.935
Non‐Hispanic white	69	57%	52	43%	
African American/Black	78	57%	60	43%	
Age at diagnosis (years)					<0.001
<50	23	82%	5	18%	
50–59	38	70%	16	30%	
60–69	67	50%	66	50%	
70+	19	43%	25	57%	
Median (range)	61 (25–77)		65 (41–79)		
Education					0.086
Less than high school	4	33%	8	67%	
High school/ GED	28	53%	25	47%	
Some college/2 year degree	59	54%	50	46%	
Four‐year college degree	32	73%	12	27%	
Graduate/ prof. degree	22	56%	17	44%	
Not reported	2	–	0	–	
Marital status					0.398
Married or equivalent	62	54%	52	46%	
Widowed	12	44%	15	56%	
Divorced or separated	34	60%	23	40%	
Never married	37	63%	22	37%	
Not Reported	2	–	0	–	
Income (household)					0.229
<$20,000	39	55%	32	45%	
$20,000–39,999	22	55%	18	45%	
$40,000–59,999	23	58%	17	43%	
$60,000–79,999	15	50%	15	50%	
≥$80,000	32	70%	14	30%	
Not reported	16	–	16	–	
Current insurance—Medicare	73	51%	71	49%	0.020
Current insurance—Medicaid	32	55%	26	45%	0.745
Current insurance—Private	90	60%	61	40%	0.319
Time from diagnosis to interview (months)					0.114
Median (IQR)	25 (19–34)		23.5 (18–31)		
Range	5–49		6–49		
*Cancer diagnosis and treatment*					
SEER summary stage					<0.001
Local	128	70%	56	30%	
Regional	17	30%	39	70%	
Distant	0	0%	14	100%	
Unstaged	2	–	3	–	
Cancer surgery					0.197
Yes	130	55%	106	45%	
No	14	70%	6	30%	
Not reported	3	–	0	–	
Radiation therapy					<0.001
Yes	37	35%	70	65%	
No	108	73%	40	27%	
Not reported	2	–	2	–	
Hormone therapy					0.874
Yes	6	55%	5	45%	
No	139	57%	105	43%	
Not reported	2	–	2	–	
Immunotherapy					0.434
Yes	2	40%	3	60%	
No	142	57%	105	43%	
Not reported	3	–	4	–	
Chemotherapy					<0.001
Yes	16	17%	79	83%	
No	128	80%	33	20%	
Not reported	3	‐	0	‐	
In treatment at enrollment					0.582
Yes	9	7%	9	8%	
No	127	93%	97	92%	
Not Reported	11	–	6	–	
Survey method of completion					0.774
Online	18	12%	14	13%	
Interviewer administered	12	8%	12	11%	
Written	117	80%	86	77%	

Table 2 describes self‐reported medical history and health behaviors that may be associated with QOL, stratified by grade. The vast majority of women were obese, although this did not differ by grade, and the majority also reported a history of hypertension. A history of diabetes and high cholesterol were also prevalent in this cohort. Current smoking status, alcohol use, and physical activity did not vary by grade.

**TABLE 2:** Select medical history and health behavior variables for women diagnosed with endometrial cancer.

	Low grade		High grade		*p*‐value
	*N*	Row %	*N*	Row %	
Total	147	57%	112	43%	
*Medical history*					
Current BMI					0.384
Underweight or normal	18	55%	15	45%	
Overweight	17	46%	20	54%	
Obese	106	58%	76	42%	
Not reported	6	–	1	–	
Median (range)	34.9 (15.1–69.4)		33.2 (19.0–69.0)		
Diabetes					0.697
Yes	47	58%	34	42%	
No	97	55%	78	45%	
Not reported	3	–	0	–	
High blood pressure					0.094
Yes	103	60%	69	40%	
No	41	49%	43	51%	
Not reported	3	–	0	–	
Stroke					0.118
Yes	6	38%	10	63%	
No	138	58%	102	43%	
Not reported	3	–	0	–	
High cholesterol					0.242
Yes	71	60%	47	40%	
No	73	53%	65	47%	
Not reported	3	–	0	–	
COPD or emphysema					0.291
Yes	9	45%	11	55%	
No	135	57%	101	43%	
Not reported	3	–	0	–	
Arthritis					0.549
Yes	73	54%	61	46%	
No	71	58%	51	42%	
Not reported	3	–	0	–	
Thyroid disease					0.197
Yes	31	65%	17	35%	
No	113	54%	95	46%	
Not reported	3	–	0	–	
Depression					0.137
Yes	34	65%	18	35%	
No	110	54%	94	46%	
Not reported	3	–	0	–	
*Health behaviors*					
Smoking status					0.305
Never	89	60%	60	40%	
Former	43	49%	44	51%	
Current	8	57%	6	43%	
Not reported	7	–	2	–	
Physical activity in past 4 weeks					0.710
Yes	97	56%	76	44%	
No	48	59%	34	41%	
Not reported	2	–	2	–	
Alcohol in past 4 weeks					0.912
Yes	54	56%	42	44%	
No	90	57%	68	43%	
Not reported	3	–	2%	–	

**FIGURE 1:**
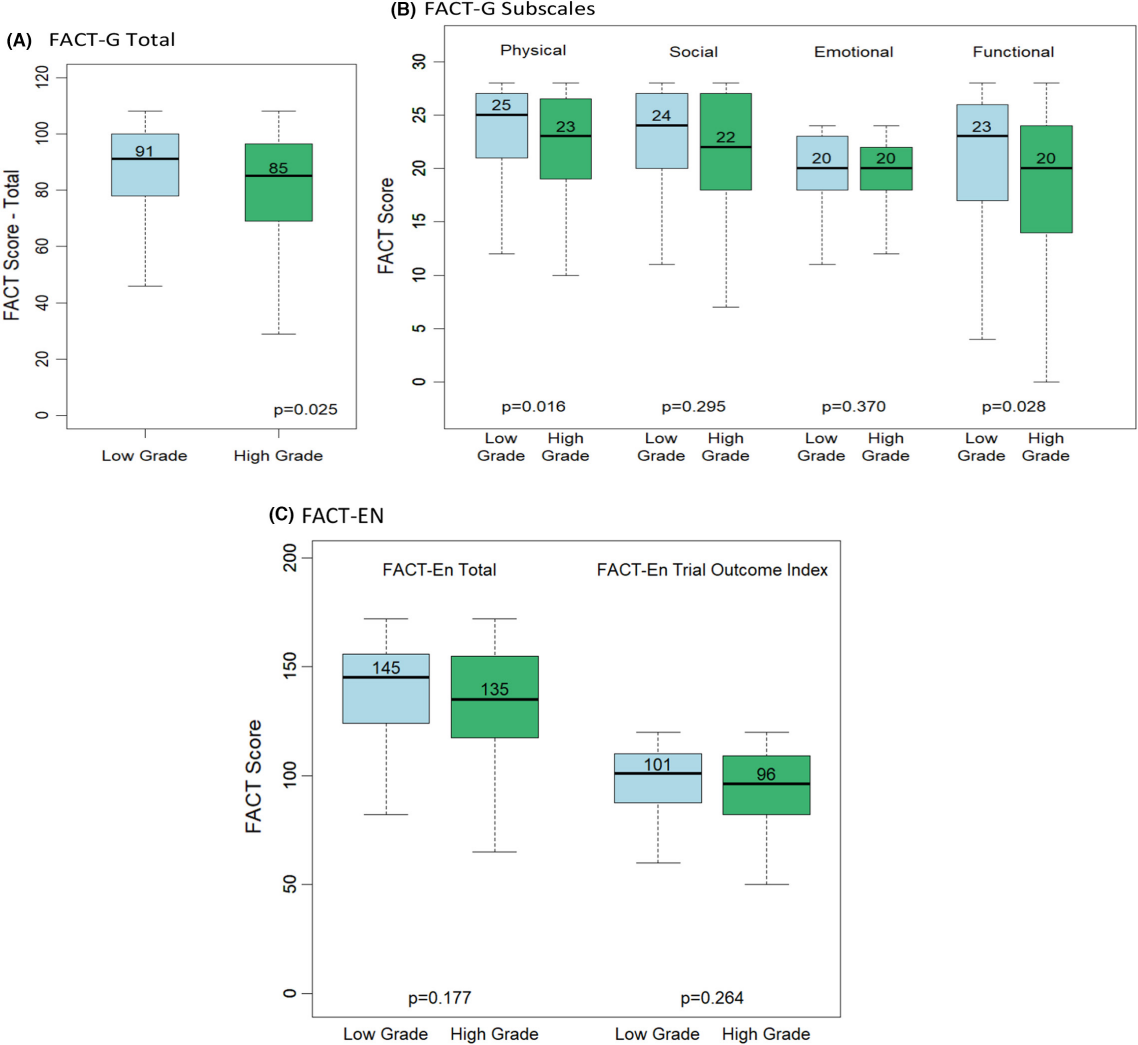
FACT scores by grade category. Box plots of FACT‐G total score (A), FACT‐G Subscales (B) and FACT‐En total score (C) stratified by grade. The dark horizontal lines indicate the overall median and the dotted lines and hashtags represents the confidence interval around the median. (A) A clinically and statistically significant difference between overall QOL scores, with women with low‐grade EC reporting better QOL compared to women with high‐grade EC. (B) Further examination of the components of the FACT‐G total and indicates statistically significant differences in physical and functional scores between women with low‐ and high‐grade disease. (C) Utilizes the FACT‐En tool to quantify QOL and shows no difference by grade.

The association between QOL (as measured by FACT‐G total scores and FACT‐G subscales) and grade is shown in Figure 1. Figure 1A depicts the difference in FACT‐G total scores, with a median score of 91 for women with low‐grade disease compared to 85 for women with high‐grade disease, which represents a clinical and statistically significant difference (*p* value = 0.025). Figure 1B separates the FACT‐G into physical, social, emotional, and functional subscales. Women with high‐grade disease report lower median physical scores compared to their low‐grade counterparts (23 vs. 25, *p* value = 0.016) and lower functional scores (20 vs. 23, *p* value = 0.028), while their emotional scores were equivalent (both = 20, *p* value = 0.37). The social subscale scores are lower for women with high‐grade disease compared to those with low‐grade disease, but not significantly so (*p* value = 0.295). Figure 1C shows no difference by grade when examining the FACT‐En scale as the QOL outcome. Cronbach's alpha for FACT‐G total was 0.91, similar to what has been reported from a meta‐analysis of this tool (0.88) and for FACT‐En was 0.86, similar to the dataset from which these measures were developed (FACT‐En, 0.91; data not shown).^20^, ^21^ These measures of internal consistency are in the good to excellent range.^15^, ^16^

Additional analyses into factors associated with FACT‐G scores are shown in Table 3. In univariate analyses, African American self‐reported race, never married, and lower household income were all associated with lower QOL scores (*p* values = 0.004, 0.008, and <0.001, respectively). Women with more education had higher FACT‐G scores than their counterparts with less education (*p* value<0.001). Private insurance was associated with higher FACT‐G total scores (*p* value = 0.002) while those with Medicaid or other/no insurance had lower scores compared with those on Medicare (*p* values = 0.051 and 0.007, respectively). Women with high‐grade disease had lower FACT‐G scores compared to women with low‐grade disease (*p* value = 0.039), as did those with regional disease compared to local disease (*p* value = 0.009) and receipt of radiation therapy (*p* value = 0.001). Women reporting diabetes or depression also had lower FACT‐G scores than those without (*p* values = 0.01 and 0.004, respectively) as did those with more comorbid conditions (*p* value = 0.004). Those who reported physical activity within the past month had higher FACT‐G scores than those who did not (*p* value = 0.013). In a multivariable model adjusted for all statistically significant variables identified in the univariate analyses, higher education was associated with higher FACT‐G scores (adjusted *p* value = 0.014) lower household income, other (including no) insurance, receipt of radiation therapy, and depression were all associated with lower FACT‐G scores (*p* values = 0.02, 0.004, 0.033 and 0.011, respectively).

**TABLE 3:** Association between select demographic, clinical, and health behavior variables and FACT‐G total.

	Univariate				Multivariable*			
	Estimate	LowerCL	UpperCL	*p*‐value	Estimate	LowerCL	UpperCL	*p*‐value
Race								
Non‐Hispanic White	Ref.				Ref.			
African American/Black	−6.5	−10.9	−2.2	**0.004**	−2.8	−7.8	2.1	0.261
Age at diagnosis	0.5	−2.1	3.1	0.694				
Education	4.6	2.7	6.6	**<0.001**	2.7	0.5	4.9	**0.015**
Marital status								
Married or equivalent	Ref.				Ref.			
Widowed	−7.6	−15.5	0.2	0.055	0.3	−8.2	8.7	0.950
Divorced or separated	−3.6	−9.3	2.1	0.215	2.4	−4.0	8.8	0.456
Never married	−7.6	−13.2	−2.1	**0.008**	2.7	−3.9	9.2	0.425
Household income (decreasing)	−4.3	−5.7	−2.9	**<0.001**	−2.4	−4.5	−0.3	**0.025**
Current insurance–Medicare	−3.1	−7.6	1.4	0.172	4.1	−1.0	9.1	0.112
Current insurance–Medicaid	−8.3	−13.5	−3.1	**0.002**	−0.4	−7.6	6.8	0.916
Current insurance–Private	7.8	3.4	12.3	**0.001**	2.3	−4.2	8.9	0.482
Histology								
Low grade	Ref.				Ref.			
High grade	−4.7	−9.1	−0.2	0.039	−1.4	−7.4	4.7	0.653
SEER summary stage								
Local	Ref.				Ref.			
Regional	−7.1	−12.4	−1.8	**0.009**	−4.8	−10.9	1.3	0.121
Distant	−8.3	−17.6	1.0	0.080	−7.2	−17.3	2.8	0.155
Cancer surgery	1.6	−7.4	10.7	0.720				
Radiation therapy	−7.6	−12.0	−3.2	**0.001**	−5.8	−10.9	−0.6	**0.027**
Hormone therapy	−8.1	−19.0	2.7	0.140				
Immunotherapy	0.1	−15.2	15.5	0.989				
Chemotherapy	−5.2	−9.7	−0.7	**0.023**	0.9	−5.7	7.4	0.794
Current BMI								
Underweight or normal	Ref.							
Overweight	−1.1	−9.5	7.3	0.799				
Obese	−3.6	−10.1	2.9	0.281				
Diabetes	−6.3	−11.0	−1.5	**0.010**	−0.4	−6.0	5.2	0.883
High blood pressure	−2.0	−6.7	2.8	0.416				
Stroke	−6.6	−15.3	2.1	0.138				
High cholesterol	−0.7	−5.1	3.8	0.768				
COPD or emphysema	−2.6	−10.7	5.5	0.526				
Arthritis	−3.6	−8.0	0.8	0.108				
Thyroid disease	−2.0	−7.6	3.7	0.491				
Depression	−7.9	−13.3	−2.5	**0.004**	−8.0	−14.2	−1.8	**0.012**
Comorbidity count	−1.7	−2.9	−0.6	**0.004**	−0.6	−2.3	1.0	0.463
Smoking status								
Never	Ref.							
Former	−2.4	−7.3	2.4	0.323				
Current	−4.4	−14.3	5.5	0.384				
Physical activity in past 4 weeks	6.1	1.3	10.8	**0.013**	4.3	−0.4	8.9	0.075
Alcohol consumption in past 4 weeks	8.5	4.0	12.9	**<0.001**	4.1	−0.6	8.8	0.086
In treatment at enrollment	−2.6	−11.0	5.9	0.551				

* Adjusted for variables significant at *p* < 0.05 in univariate analyses, highlighted in bold.

## 4 DISCUSSION

Women diagnosed with EC often experience favorable outcomes; however, EC is a heterogenous disease with different subtypes that impact prognosis, particularly disease grade. Here, we reported QOL estimates from a population‐based study of women who have been diagnosed with EC, utilizing the FACT‐G and FACT‐En tools. Women with high‐grade EC reported overall lower QOL compared to women with low‐grade disease as measured by the FACT‐G, although they had similar EC‐specific QOL indicators, as measured by the FACT‐En. The differences seen in the FACT‐G scores represent minimal clinically important differences and are seen primarily in the physical and functional realms. It is not totally unexpected to see these realms together, given that the former focuses on physical symptoms, such as having nausea or pain, and the latter focuses on functional outcome, such as the ability to work. Similarly, although no differences were identified, the emotional and social realms may be related, with the emotional realm focused on inward feelings (sadness, nervousness, worry) and the social realm measuring personal connections (support from friends, family, partner). It is possible that the women who participated in this study did so because of higher levels of social support, regardless of grade of disease. It is also possible that emotional and social well‐being does not have long‐lasting negative affects after an EC diagnosis, or that women feel more supported during this time, whereas the physical and functional changes may take longer, or may never, resolve. After adjustment for other factors associated with QOL as measured by FACT‐G, we found that socio‐economic measures (lower income and less education), receipt of radiation, and clinical depression were all independently associated with lower QOL in this cohort, similar to the findings reported for all gynecologic cancers by Glasspool et al.^10^

Lower income and lower education were associated with lower QOL scores. Alitic et al. found cancer survivors with lower income were more likely to experience financial hardship and as the cost of cancer therapy continues to rise this increases the economic burden on cancer patients and survivors.^22^, ^23^ In effect, cancer survivors may seek treatment options that are not as efficacious due to lower cost, delay care, or forego medical therapy due to cost.^22^ While this prior work was not specific to EC survivors, it is likely that socioeconomic factors may have lasting consequences and thus impact their QOL.

Those who underwent radiation therapy had lower overall QOL scores, even after adjustment for grade of disease. Effects of chemoradiation in a study of women with early stage EC reported overall lower physical and role functioning scores, which in turn correlated with lower QOL scores, consistent with our findings.^24^ Also similar to our findings utilizing the FACT‐En tool to assess QOL,^25^ a recent report from Matulonis et al. examining QOL in women with locally advanced EC based on receipt of chemotherapy and radiation versus chemotherapy alone also noted no clinically meaningful differences in QOL using this scale.^26^ A systematic review of patient‐reported outcomes in EC revealed the FACT‐En tool is not widely used.^12^ A future extension of this work could better define women at greatest risk of poorer QOL based on type of radiation treatment, as well as additional refinement of EC‐specific measures.

Our study reported an association between depression and lower QOL. Cancer patients were more likely to screen positive for depressive symptoms, and therefore experience lower QOL scores which has been associated with lower overall survival.^9^, ^26^ A hospital‐based study assessing prevalence of depression showed rates of depression in gynecologic cancer patients (ovarian, cervical, gestational trophoblastic tumors, and ECs), (*n*_total_ = 149; 20 with EC) as high as 13%, more strongly associated with low income, cervical cancer, radiation therapy, and poor performance status.^26^ A longer‐term study of women with early‐stage EC noted higher prevalence of anxiety and depression even at 4.5 years of follow‐up compared to population‐based norms.^27^ This highlights the need for continued assessment of mental health status in this population.

Obesity is a prevalent and modifiable risk factor for development of EC, and may impact QOL, although it was not independently associated with QOL in our cohort. This is in contrast to literature that suggests lower QOL is associated with higher BMI.^28^ Similarly, there was no association between higher levels of physical activity and increased QOL, in contrast to findings in EC survivors by Courneya et al., who reported EC survivors who met public health guidelines for exercise had a higher QOL score compared to those who did not meet the recommended guidelines.^29^ While the findings are still mixed with respect to physical activity and obesity, a recent population‐based study noted that nearly as many EC survivors died of other causes such as cardiovascular and cerebrovascular diseases at similar rates of succumbing to EC, suggesting that recommendations for physical activity and body size are important in this population for overall survival.^30^

There are strengths and limitations to our study that should be considered when appraising the findings. Our sample is population‐based, and includes a wide range of self‐reported demographic, socio‐economic, and behavioral factors that may influence QOL, which was measured using validated tools. We had a greater proportion of African American women in our study, and we also oversampled high‐grade EC tumors, which are responsible for the majority of morbidity and mortality from EC. A potential limitation of our study is that participants were not contacted until at least 5 months post‐diagnosis, and the median time from diagnosis to baseline interview was nearly 2 years. Thus, women with shorter survival periods were not represented in this cohort, which introduced survivorship bias. This may have particularly affected women with high‐grade EC undergoing continued treatment who were not well enough to participate or those with rapidly fatal disease. In addition, it was surprising that the FACT‐G tool identified differences by grade, but the FACT‐En did not. A recent systematic review of patient‐reported outcomes in non‐pharmacological interventions for EC survivors noted that of the 10 studies included, FACT‐G was the most commonly used tool (*n* = 4), but FACT‐En was not included in any of the studies.^31^ Continued research to determine how to best assess QOL in EC survivors is necessary to identify ways to intervene in this growing population. Even with these potential limitations, our respondents represented all stages at diagnosis, histologic types, and reported a range of QOL measures.

In conclusion, we provided evidence that women with high‐grade disease reported overall lower QOL scores, particularly in physical and functional realms, compared to women with low‐grade disease. Interestingly, the EC‐specific QOL measures did not find differences by grade, suggesting this tool did not capture additional information to tease out components of QOL in our population. While the burden of cancer in a population is often described in terms of incidence and mortality, reducing the morbidity associated with cancer survivorship is also important. Understanding these specific challenges can help clinicians address them throughout the life course after an EC diagnosis. Ultimately, it is essential to understand the individual factors that contribute to overall health related QOL in order to develop appropriate interventions in this growing population of EC survivors.

## AUTHOR CONTRIBUTIONS

**Kaitlyn Banning:** Writing – original draft (lead). **Juliana Fucinari:** Data curation (lead); project administration (supporting); writing – original draft (supporting). **Abigail Fielder:** Writing – original draft (supporting). **Julie J. Ruterbusch:** Data curation (supporting); formal analysis (lead); project administration (supporting); writing – original draft (supporting). **Jennifer Beebe‐Dimmer:** Funding acquisition (supporting); project administration (supporting); writing – original draft (supporting). **Ann Schwartz:** Conceptualization (lead); funding acquisition (lead); project administration (supporting); writing – original draft (supporting). **John J. Wallbillich:** Writing – original draft (supporting). **Michele L. Cote:** Conceptualization (lead); formal analysis (supporting); funding acquisition (supporting); methodology (lead); writing – original draft (equal).

## CONFLICT OF INTEREST STATEMENT

The authors whose names are listed immediately below certify that they have NO affiliations with or involvement in any organization or entity with any financial interest (such as honoraria; educational grants; participation in speakers' bureaus; membership, employment, consultancies, stock ownership, or other equity interest; and expert testimony or patent‐licensing arrangements), or non‐financial interest (such as personal or professional relationships, affiliations, knowledge or beliefs) in the subject matter or materials discussed in this manuscript.

## Data Availability

NA.
